# New strategy to rescue the inhibition of osteogenesis of human bone marrow-derived mesenchymal stem cells under oxidative stress: combination of vitamin C and graphene foams

**DOI:** 10.18632/oncotarget.12456

**Published:** 2016-10-04

**Authors:** Zubin Zhou, Zhengliang Xu, Feng Wang, Ye Lu, Peipei Yin, Chaolai Jiang, Yingjie Liu, Hua Li, Xiaowei Yu, Yuqiang Sun

**Affiliations:** ^1^ Department of Orthopaedic Surgery, Shanghai Jiao Tong University Affiliated Sixth People's Hospital, Shanghai 200233, China; ^2^ State Key Laboratory of Metal Matrix Composites, School of Materials Science and Engineering, Shanghai Jiao Tong University, Shanghai 200240, China

**Keywords:** osteogenesis, mesenchymal stem cell, oxidative stress, vitamin C, graphene foams

## Abstract

To rescue the oxidative stress induced inhibition of osteogenesis, vitamin C (VC) was chemically modified onto three-dimensional graphene foams (3D GFs), then their regulation on osteogenesis of human bone marrow-derived mesenchymal stem cells (BM-MSCs) was studied. Combined action of VC + GF significantly decreased H_2_O_2_-induced oxidative stress, and rescued H_2_O_2_-inhibited cell viability, differentiation and osteogenesis of BM-MSCs *in vitro*. Further studies revealed that Wnt pathway may be involved in this protection of osteogenesis. Furthermore, an *in vivo* mouse model of BM-MSCs transplantation showed that VC + GF remarkably rescued oxidative stress inhibited calcium content and bone formation. The combination of VC and GF exhibited more pronounced protective effects against oxidative stress induced inhibition of osteogenesis, compared to monotherapy of VC or GF. Our study proposed a new strategy in stem cell-based therapies for treating bone diseases.

## INTRODUCTION

The homeostasis of human bones is tightly balanced by the orchestrated activities of bone-forming osteoblasts and bone-resorbing osteoclasts. Oxidative stress, resulted from excessive reactive oxygen species (ROS) production, disrupts the homeostasis by inhibiting osteoblast function and stimulating osteoclastogenesis [1]. Chronic exposure of oxidative stress may cause bone impairments and diseases [2]. Also, oxidative stress was reported to be a major contributing factor of osteoporosis during aging [3, 4]. At the molecular level, ROS, including superoxide anion, hydroxyl radical, hydroxyl ion, nitric oxide (NO) and hydrogen peroxide, react with DNA, protein and lipids inside cells, which further induces cytokine-mediated pro-inflammation. Meanwhile, excessive ROS are able to react with NO forming peroxynitrite that in turn could oxidize tetrahydrobiopterin, a cofactor of endothelial nitric oxide synthase (eNOS), and result in its decoupling from eNOS, leading to further elevated ROS, as well as decreased NO productions.

Mesenchymal stem cells (MSCs) are favorable candidates for regenerative medicine and tissue engineering in the clinical management of bone-related diseases. MSCs can undergo differentiation and give rise to various other cell types, including osteoblasts, muscle cells, chondrocytes and adipocytes [5–8]. Bone marrow-derived mesenchymal stem cells (BM-MSCs) exhibit high differentiating potential into the osteoblast lineage [9], which can be potentially used for cell transplantation during clinical treatment of bone diseases. In an earlier clinical trial, the therapeutic potential of BM-MSCs was clearly demonstrated in children suffering from osteogenesis imperfecta [10].

In living organisms, vitamin C (VC) is a potent reducing reagent, which is able to quickly scavenge various ROS. VC has been studied for the treatment of different bone diseases [11–14]. Three dimensional graphene foams (3D GFs) is a new type of nanomaterial, and its unique physical and chemical properties have sparked increasing interests since its discovery in 2004 [15]. It was reported that graphene-based materials may serve as a free radical scavenger to reduce oxidative stress in tissue engineering [16–18], which may be associated with the sp^2^-carbon network in these graphene materials.

In our current study, we aimed to develop a new strategy using MSC-based stem cell therapy for bone diseases, by combining VC and 3D GFs. The effect of the new strategy was examined both in *in vitro* cell culture and *in vivo* animal models.

## RESULTS

### Modification of GFs with VC

**Figure 1:**
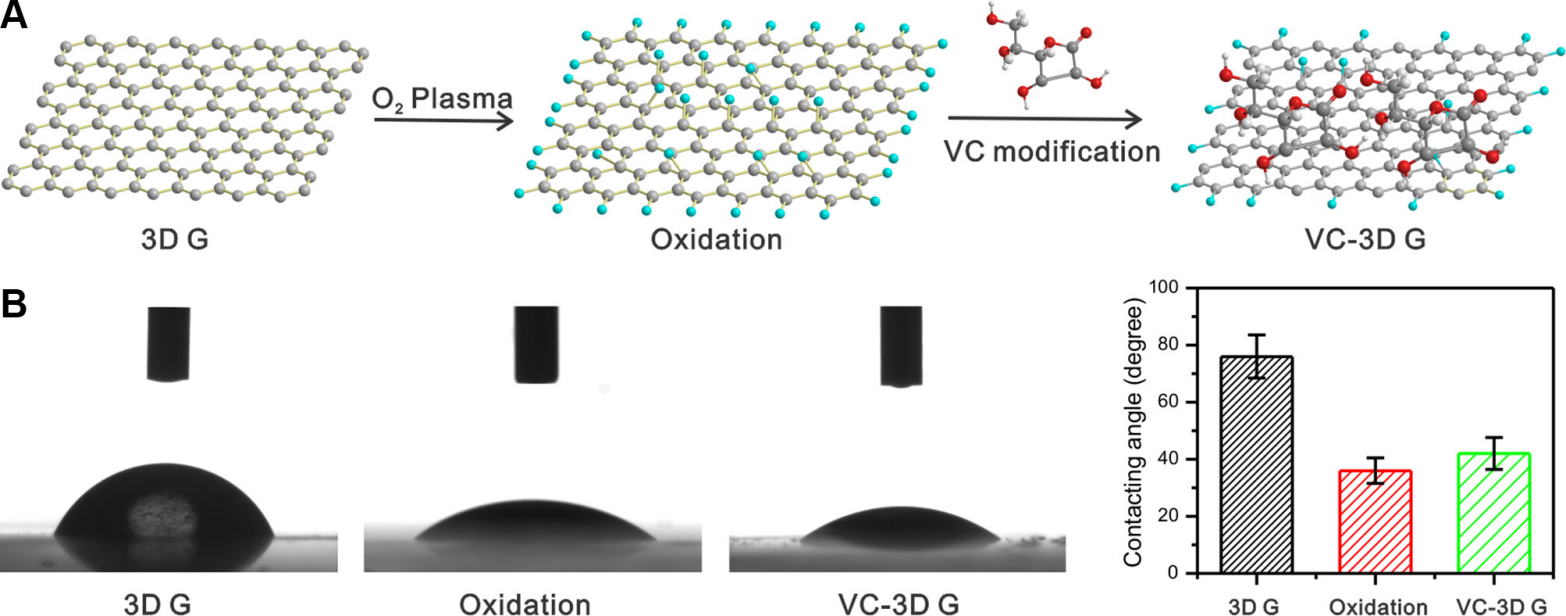
(**A**) A schematic of 3D GF treated with O_2_ plasma and VC modification. (**B**) The contact angles of 3D GF pristine, O_2_ plasma treated 3D G and VC modified 3D GF.

As illustrated in Figure 1A, GFs were exposed with O_2_ plasma and sequentially modified with VC. The contact angle of pristine graphene film was approximately 76°C, which decreased to 36°C after O_2_ plasma treatment (Figure 1B). The increased wettability of graphene with O_2_ plasma exposure was ascribed to the introduction of functional groups containing oxygen [25]. VC is a widely used biocompatible agent to reduce graphene oxide [26–29]. Hence, the content of functional groups containing oxygen on the surface of graphene was decreased, resulting in a slightly increased contact angle.

**Figure 2:**
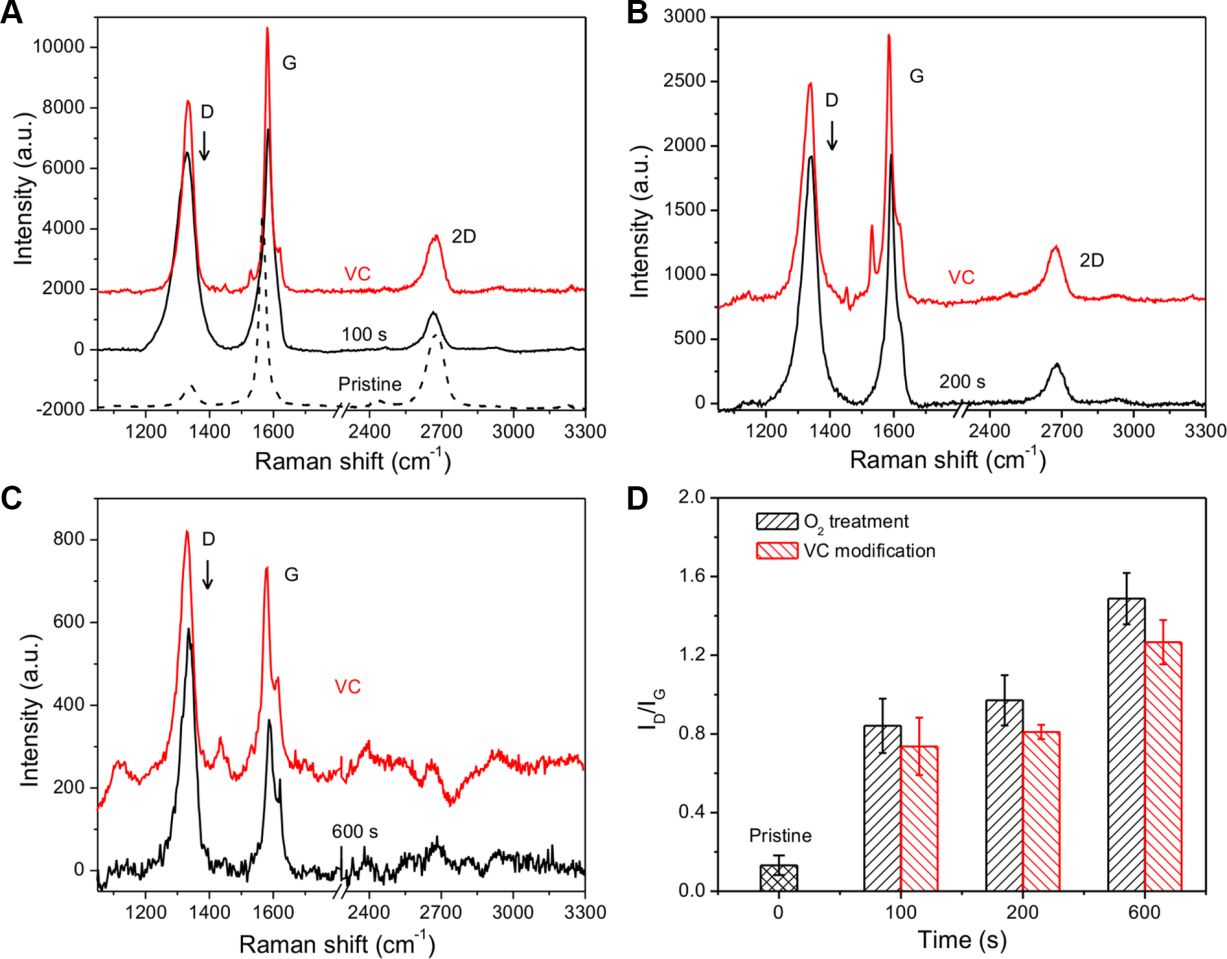
Raman spectra of 3D GF, O_2_ plasma treated 3D GF and VC modified 3D GF The 3D GF was treated by O_2_ plasma for (**A**) 100 s, (**B**) 200 s and (**C**) 600 s. (**D**) The intensity ratio of D peak and G peak as a function of the exposure time to O_2_ plasma and VC modification.

To clarify the structure change of graphene, Raman spectroscopy was employed to characterize the graphene and its derivatives. Figure 2A shows the development of Raman spectrum exposed to O_2_ plasma and VC modification. The Raman spectrum from pristine graphene indicated that graphene had the few-layered structure. A weak D peak associated with disorder was observed, showing excellent uniformity in the sample of pristine graphene. Exposing the O_2_ plasma for 100 s has made the D peak react strongly at approximately 1340/cm (Figure 2B). With increasing exposure time, the Raman spectrum showed systematic developments in position and peak intensity (*I*) until exposure of 600 s (Figure 2C), where the intensity of D and G were tremendously decreased and the 2D peak disappeared compared to background signals. We hereby define the peak intensity as the peak height. The Raman spectrum probes sites of sp^2^ through excitation of π-π stacking, and amorphous carbon atom with bonding of sp^3^ generates weaker spectrum [30]. I(D)/I(G) can then be used to evaluate the degree of disorder related to the crystalline cluster diameter of the graphene nanosheet [31]. Hence, as shown in Figure 2D, in O_2_ plasma exposed graphene, I(D)/I(G) was increased with longer exposure time, indicating a reduction in the sp^2^ ordered ring number. In contrast, I(D)/I(G) declined after VC modification, which demonstrated the disordered sp^3^ bonding was partially reduced to sp^2^ state with the introduction of VC molecules.

Detailed compositional analysis of VC-modified GF was conducted and compared with pure VC and O_2_ plasma-treated GF by X-ray photo-electron spectroscopy (XPS) (Supplementary Figure S1). The XPS of GF displayed 4 different carbon bonds: sp^2^ carbon in C–C (284.9 eV, carbon in C–O (285.8 eV), carbonyl carbon in C = O (287.6 eV), as well as carboxylate carbon in C(O) = O (288.7 eV). After GF was modified and reduced with VC, the peak of C–C at 284.4 eV was tremendously enhanced, whereas the oxidized carbon species peaks were decreased. In addition, the relatively strong signals at 285.6 eV and 287.3 eV were contributed by the C = O and C–O of VC, suggesting successful immobilization of VC molecules onto the graphene.

### Human BM-MSCs growth under different conditions

**Figure 3:**
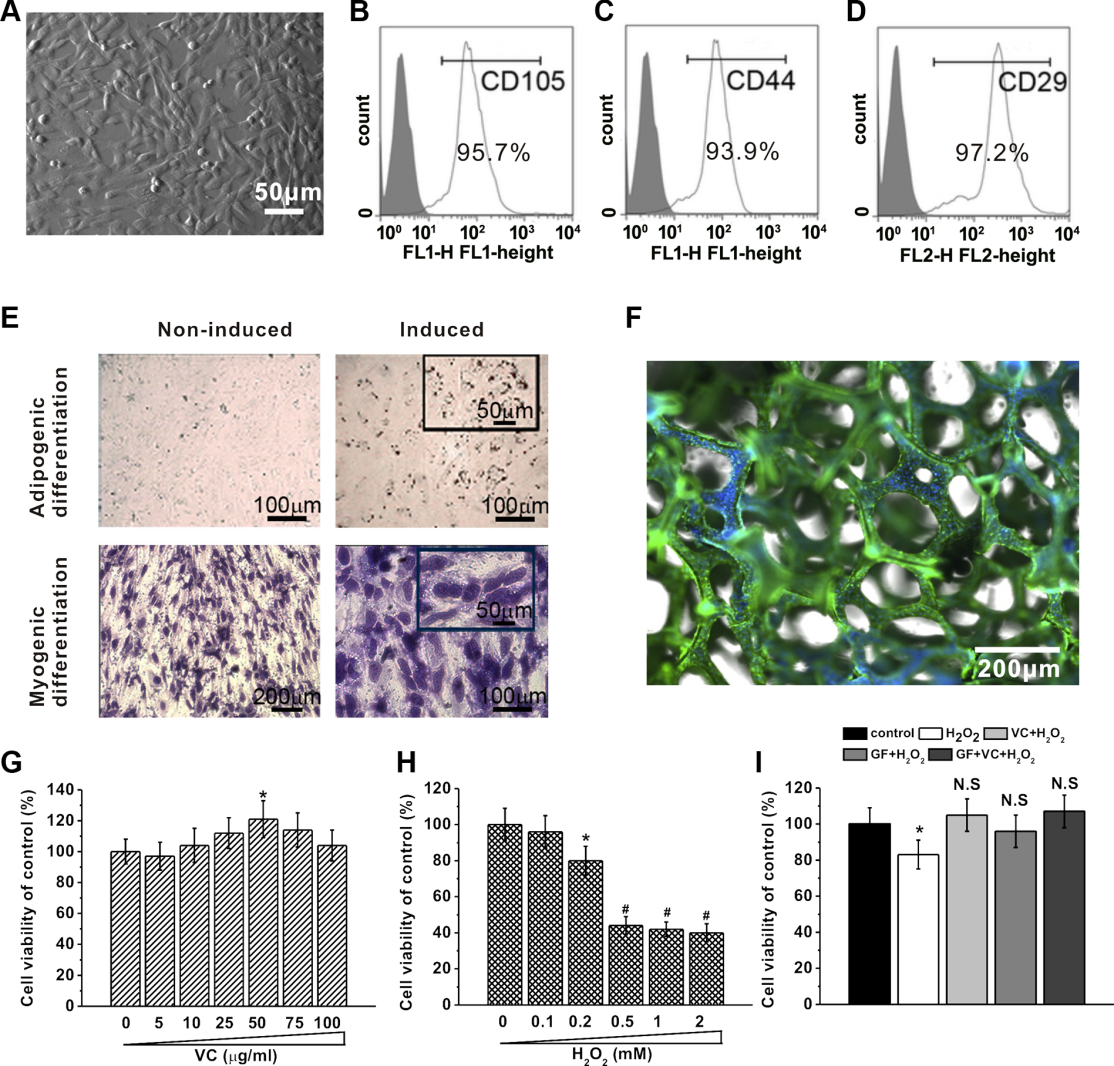
BM-MSCs growth under different concentrations of VC, H_2_O_2_ treatment or on GF substrates (**A**) Representative image of BM-MSCs 5 days after seeding. (**B**–**D**) Characterization of BM-MSCs by flow cytometry. The majority of the cells are CD105^+^, CD44^+^ and CD29^+^, which are typical characteristic phenotypes of BM-MSCs. (**E**) BM-MSC can be differentiated into adipogenic and myogenic lineages. Adipogenic differentiation was characterized by Oil Red O staining, while myogenic differentiation was evidenced by the formation of myotubes stained with crystal violet. (**F**) Representative image of immunostaining of BM-MSCs on GF scaffold, stained by anti-β-tubulin (green) and DAPI for nucleus (blue). Effects of different concentrations of VC (5 to 100 μg/ml, 5 days) (**G**) and H_2_O_2_ exposure (0.1 to 2 mM, 24 hours) (**H**) on cell viability of BM-MSCs cultured for 5 days, measured by MTT assay. (**I**) Cell viability of BM-MSCs in the five experimental groups. Data were presented as mean ± SEM. *p < 0.05 vs control.

Human BM-MSCs were isolated, exhibiting their typical MSC spindle-shape (Figure 3A). Flow cytometric analysis clearly showed that the cells isolated in our study were positive for CD105 (Figure 3B), CD44 (Figure 3C) and CD29 (Figure 3D), all of which are surface markers characteristic of MSCs. Further experiments showed that the isolated cells preserved their pluripotency, as evident by differentiation into myogenic (Figure 3E, left) and adipogenic (Figure 3E, right) lineages. In addition, the human BM-MSCs attached and grew well on the GF surface, as illustrated by a representative immunostaining image (Figure 3F) in which the cells were labeled with anti-β-tubulin antibody (green) and DAPI for the cell nucleus (blue).

Then, viability of the BM-MSCs was measured using the MTT assay. Cells treated with VC were viable, and VC at the dose of 50 μg/ml enhanced cell viability (Figure 3G. As an oxidative stress inducer, H_2_O_2_ decreased cell viability and such suppression was significant when the concentration was higher than 0.2 mM (Figure 3H). 25 μg/ml VC and 0.2 mM H_2_O_2_ were used in the following experiments since they caused significant changes in term of cell viability. The cytotoxicity of 0.2 mM H_2_O_2_ was significantly rescued, and the cell viability was restored to normal level by 25 μg/ml VC, GF and VC + GF (*p* > 0.05, Figure 3I), strongly indicating their antioxidant effect.

### GF, VC and GF+VC successfully attenuated H_2_O_2_-induced oxidative stress in human BM-MSCs

**Figure 4:**
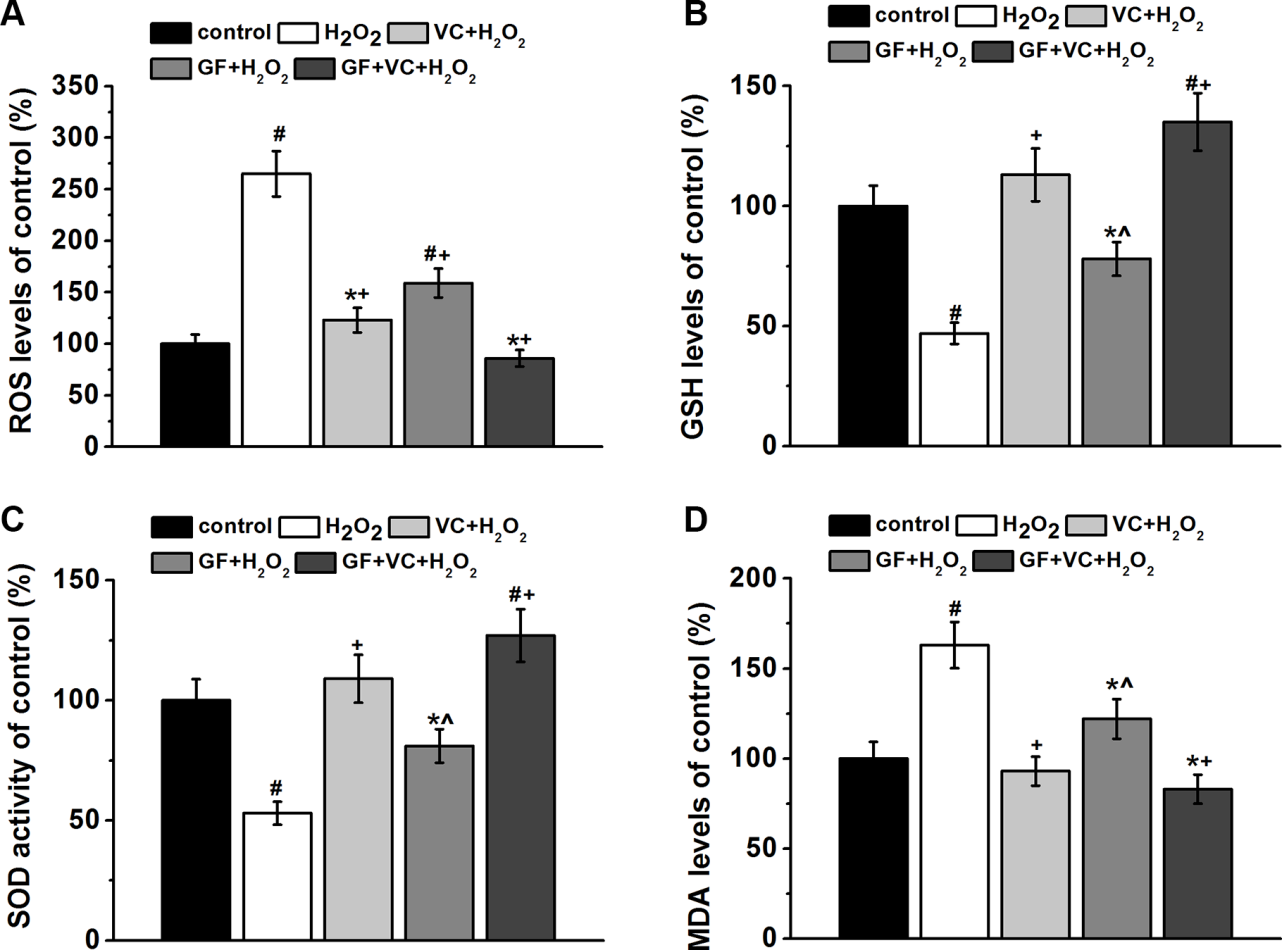
GF, VC and GS + VE successfully attenuated 0.2 mM H_2_O_2_-induced oxidative stress in BM-MSCs Oxidative stress was characterized by determining the intracellular ROS levels (**A**), GSH levels (**B**), SOD activity (**C**) and MDA levels (**D**) in the cultures for 7 days. Data were presented as mean ± SEM. *p < 0.05 and ^#^*p* < 0.01 vs control, ^*p* < 0.05 and ^+^*p* < 0.01 vs H_2_O_2_ treatment group.

Meanwhile, the effects of GF and VC on H_2_O_2_-induced oxidative stress were examined in human BM-MSCs. 0.2 mM H_2_O_2_ induced the ROS level in the culture to about 260% of the control without treatment. The H_2_O_2_-induced ROS increase was attenuated by 25 μg/ml VC (*p* < 0.05) and GF (*p* < 0.05) (Figure 4A), while such attenuation was more pronounced to a level of 82% by the co-treatment of VC + GF (Figure 4A). In addition, changes in the endogenous non-enzymatic antioxidant glutathione (GSH) level confirmed the rescue effect of VC and GF: the GSH level was inhibited to about 48% by the treatment of 0.2 mM H_2_O_2_, which was significantly rescued by VC (25 μg/ml, *p* < 0.05, Figure 4B) and GF (Figure 4B). Consistently, such rescue effect was even more pronounced in the VC + GF group (Figure 4B). Moreover, superoxide dismutase (SOD) activities confirmed that 0.2 mM H_2_O_2_ induced oxidative stress (*p* < 0.05), which was then significantly rescued by VC, GF, and VC + GF (*p* < 0.05, Figure 4C). Not surprisingly, malondialdehyde (MDA) level, another marker of oxidative stress, was significantly upregulated by the treatment of 0.2 mM H_2_O_2_, which was again significantly inhibited by either VC or GF, showing even more pronounced effect in the VC + GF group (Figure 4D).

### Differentiation of human BM-MSCs

**Figure 5:**
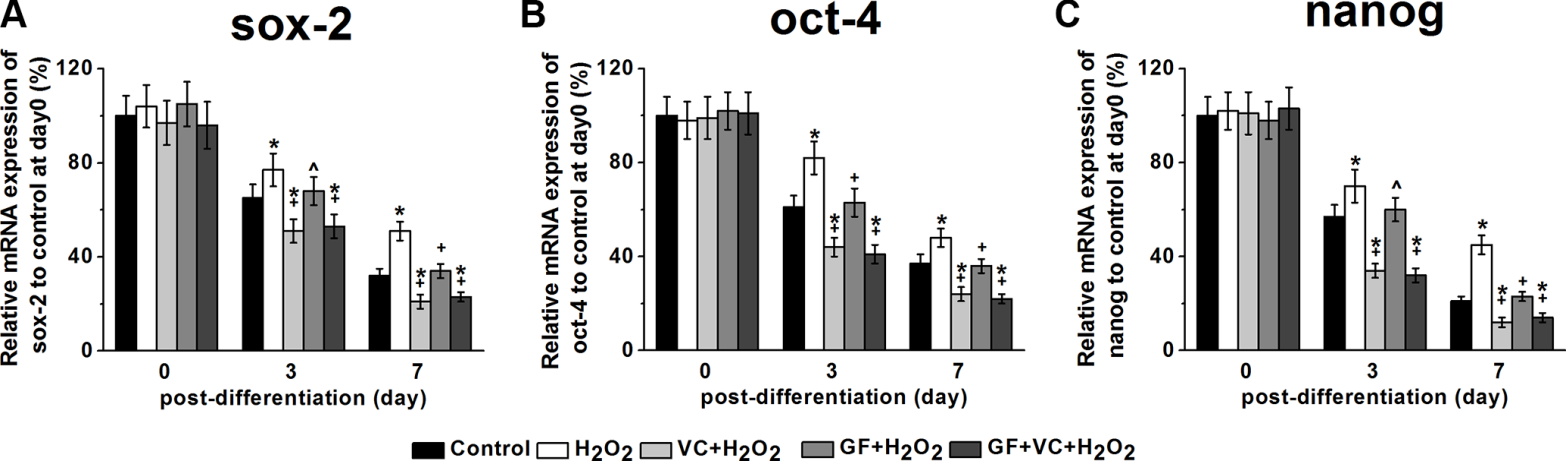
*Sox-2* (A), *oct-4* (B) and *nanog* (C) mRNA expressions of the MSCs in the five experimental groups during differentiation mRNA expressions were measured by RT-PCR. The data were normalized to their corresponding control at post differentiation day 0 and presented as mean ± SEM. *p < 0.05 vs control, ^*p* < 0.05 and ^+^*p* < 0.01 vs H_2_O_2_ treatment group.

Sox-2, oct-4, and nanog are stem cell markers, whose downregulation correlates with the loss of pluripotency and the beginning of subsequent differentiation steps. We examined their respective levels in the culture after different treatments during differentiation in the culture medium for osteogenic differentiation in the first 7 days. RT-PCR showed that the *sox-2* mRNA level decreased with time in the control, as the cells were being differentiated (Figure 5A). However, the reduction of the sox-2 level was inhibited after the treatment of 0.2 mM H_2_O_2_ compared to the control (Figure 5A), which can be rescued by VC, GF and VC + GF treatments (Figure 5A). These results suggested that the H_2_O_2_-impaired differentiation was rescued with the application of VC, GF and VC + GF. Similarly, such rescued differentiation in the VC, GF and VC + GF group was also confirmed by the changes in levels of *oct-4* (Figure 5B) and *nanog* mRNA (Figure 5C).

### Osteogenesis of human BM-MSCs

**Figure 6:**
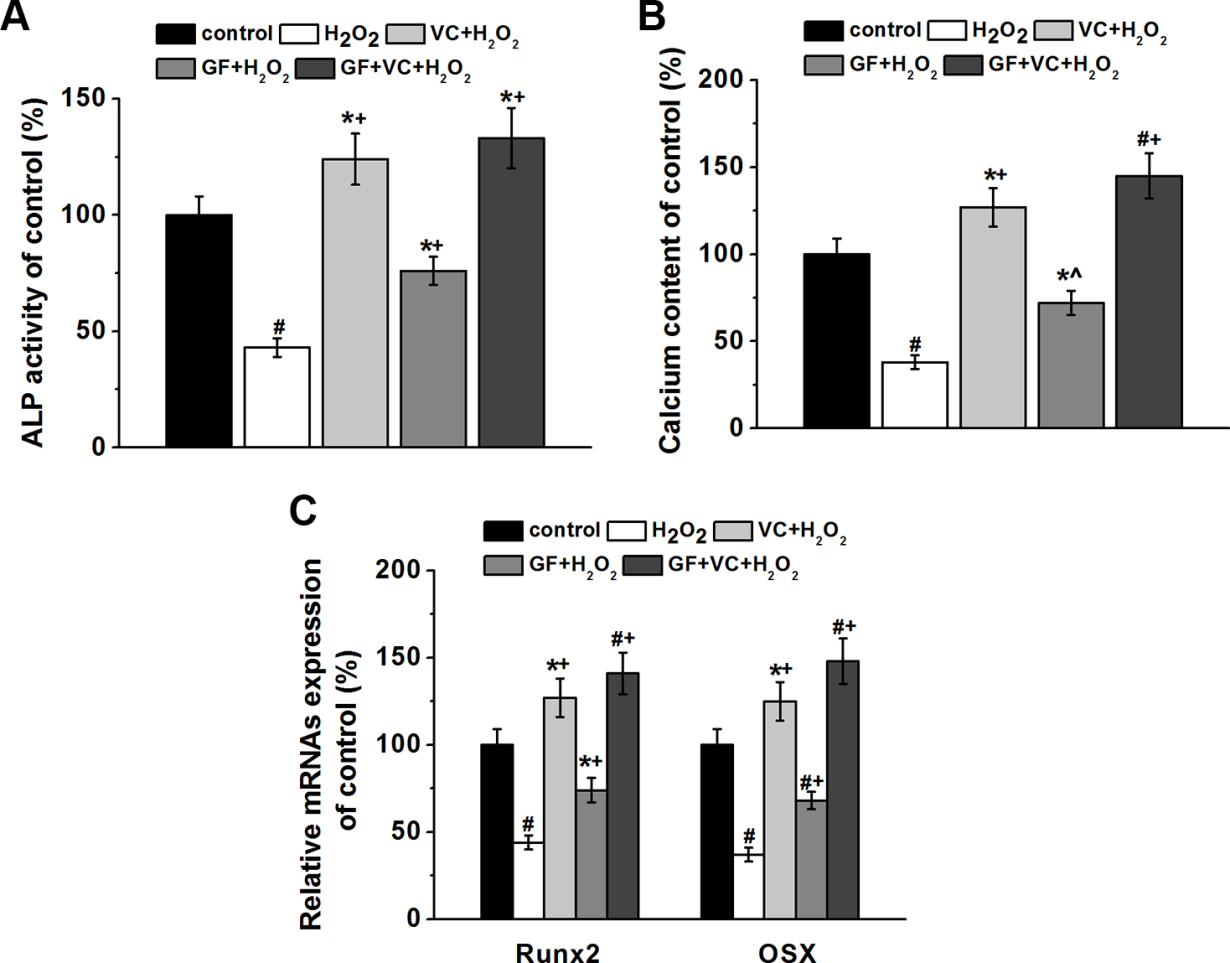
Both GF and VC protected against 0.2 mM H_2_O_2_-induced inhibition of osteogenic differentiation of BM-MSCs ALP activity (**A**) and calcium contents (**B**) of the cultures of the four experimental groups normalized to control. (**C**) Relative mRNAs expressions of *Runx2* and *Osx* in the four experimental groups, quantified by RT-PCR. Data were presented as mean ± SEM. *p < 0.05 and ^#^*p* < 0.01 vs control, ^*p* < 0.05 and ^+^*p<* 0.01 vs H_2_O_2_ treatment group.

Furthermore, we investigated the osteogenesis of human BM-MSCs in the culture directly. 0.2 mM H_2_O_2_ decreased the ALP activity to approximately 42% after 8 days of differentiation (Figure 6A). Such decrease was rescued in the VC, GF and VC + GF treatment group (*P* < 0.05, Figure 6A). Calcium contents examined on day 16 confirmed that 0.2 mM H_2_O_2_ inhibited osteogenesis, which was also successfully restored by the application of VC, GF and VC + GF (*p* < 0.05) (Figure 6B). Of note, co-treatment of VC + GF rescued the H_2_O_2_-suppressed differentiation more significantly compared to treatment of VC or GF alone (Figure 6A and 6B). *Runx2* and *Osx* are two important marker genes of osteogenesis. RT-PCR revealed that H_2_O_2_ inhibited the transcript levels of *Runx2* and *Osx* on day 7 during differentiation (*p* < 0.05, Figure 6C), and such inhibition was rescued by the application of VC, GF and VC + GF (*p* < 0.05, Figure 6C). Altogether, H_2_O_2_ inhibited the osteogenesis of human BM-MSCs, whereas VC, GF and VC + GF significantly restored H_2_O_2_-inhibited osteogenesis. In addition, the combination of VC + GF was consistently more effective than VC and GF alone.

### The Wnt pathway was involved in the protective effects of GF and VC

**Figure 7:**
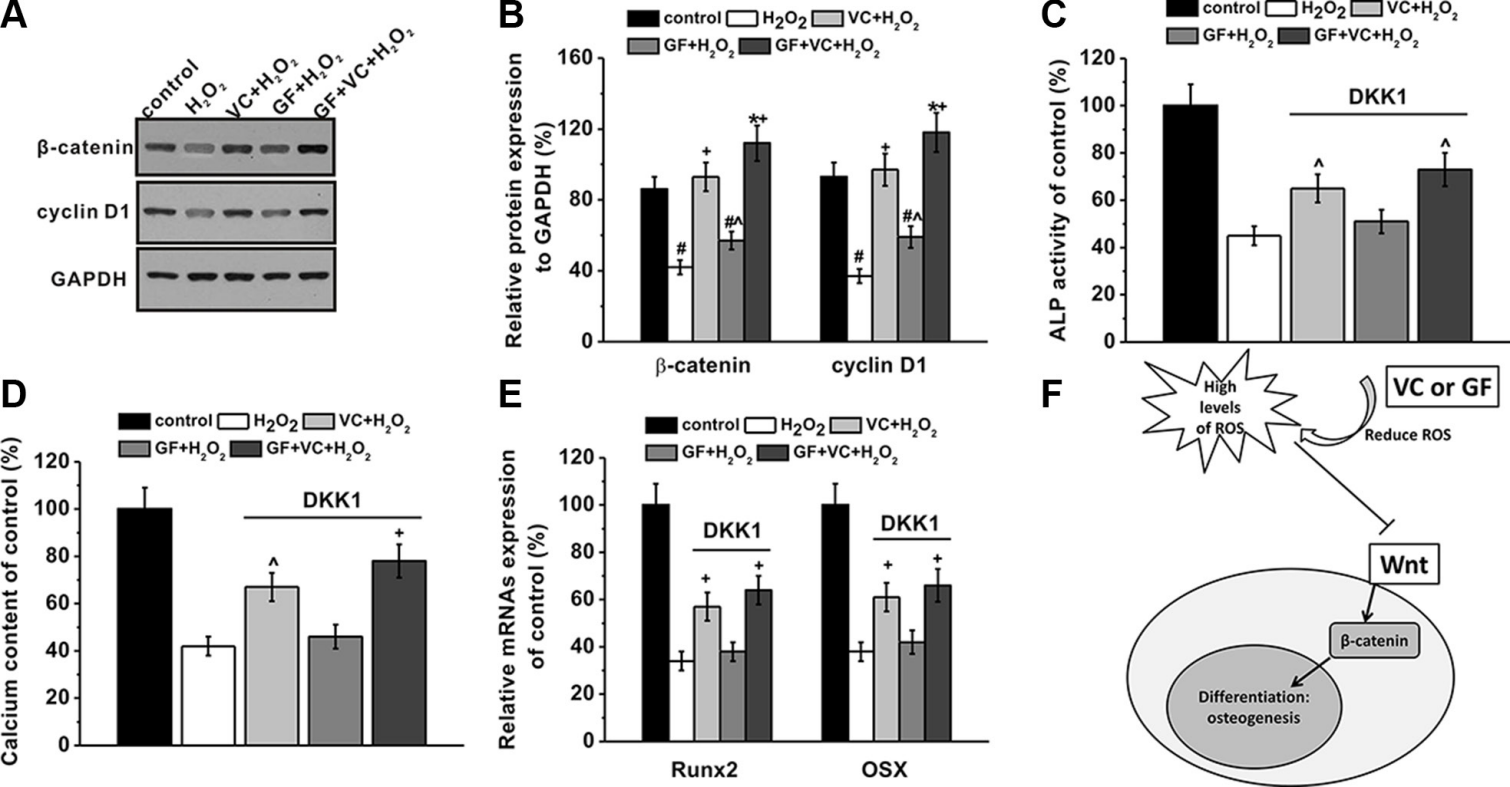
Wnt pathway was involved in the protective effects of GF and VC on the inhibition of osteogenic differentiation induced by 0.2 mM H_2_O_2_ (**A**) Western blot analysis (A) and the relative protein expression (**B**) of Wnt pathway-related regulators β-catenin and Cyclin D1 in the cultures of experimental groups. DKK-1, wnt pathway inhibitor, blocked the protective effects of VC or GF on the inhibition of osteogenic differentiation by H_2_O_2_ exposure, as evidenced by ALP activity (**C**), calcium contents (**D**) and relative mRNAs expression of *Runx2* and *Osx* (**E**). (**F**) A schematic picture showing the possible role of wnt signaling pathway. Data were presented as mean ± SEM. *p < 0.05 and ^#^*p* < 0.01 vs control, ^*p* < 0.05 and ^+^*p* < 0.01 vs H_2_O_2_ treatment group.

The Wnt pathway is involved in osteogenesis of MSCs (Figure 7F). Western blot analysis demonstrated that 0.2 mM H_2_O_2_ inhibited the expression of β-catenin and cyclin D1 (Figure 7A), and such inhibition was restored by the co-application of VC, GF and VC + GF (Figure 7A). Statistical analysis from 3 independent experiments indicated the inhibition and further restoration were both significant (Figure 7B). As a gain-of-function assay, the Wnt pathway was inhibited by a specific inhibitor DKK-1 at 0.2 μg/ml, consistent with previous study [32]. After the application of DKK-1, alkaline phosphatase (ALP) activity on day 8 was inhibited by 0.2 mM H_2_O_2_ down to about 42%, as measured in a previous experiment (Figure 6A). However, ALP activities after application of VC, GF and VC + GF were 63%, 51% and 72%, respectively (Figure 7C). This was significantly lower than those in the absence of DKK-1, which were 124%, 75% and 133%, respectively (Figure 6A). These above results suggested that the Wnt pathway was involved in the protective effects of VC, GF and VC + GF against H_2_O_2_-induced inhibition of osteogenic differentiation. Similarly, calcium content on day 16 was rescued by VC, GF and VC + GF, albeit to a markedly lower extent (Figure 7D) than in the presence of Wnt pathway function (Figure 6B). This result further confirmed that the Wnt pathway was involved in the protective effects of VC, GF and VC + GF. Such involvement of the Wnt pathway was also validated by the mRNA expression levels of *Runx2* and *Osx* (Figure 7E).

### *In vivo* osteogenesis of MSCs under different treatment

**Figure 8:**
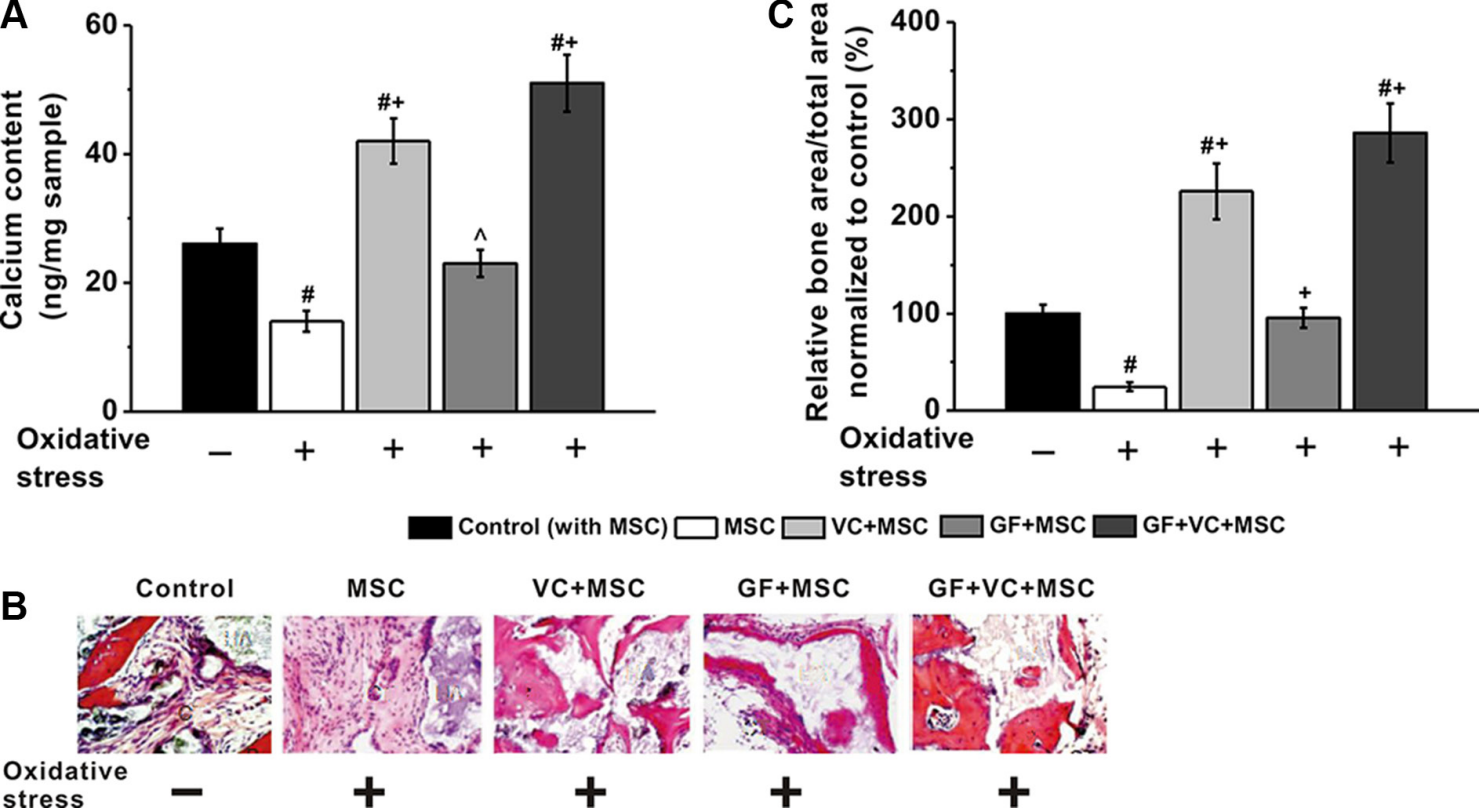
VC and GF facilitated *in vivo* osteogenesis of MSCs impaired by oxidative stress VC, GF and co-treatment of VC and GF groups enhanced calcium contents in an *in vivo* nude mice model (**A**). (**B**) Representative images of H&E staining of tissue samples in these five experimental groups. (**C**) Quantitative analysis of the amount of new bone formation in these groups. Data were presented as mean ± SEM. ^#^*p* < 0.01 vs control mice without oxidative stress, ^*p* < 0.05 and ^+^*p* < 0.01 vs mice with oxidative stress.

Furthermore, an *in vivo* mice model of osteogenesis was employed. A total of 1 × 10^6^ human BM-MSCs were injected subcutaneously into 8 weeks old male athymic nude mice. First, calcium contents were measured 2 weeks later, which was significantly lower in oxidative stress treatment group compared to control. However, such reduced calcium content was significantly rescued by treatment of VC, GF and VC + GF under oxidative stress, with VC + GF co-treatment displaying the most effective capability (Figure 8A). Besides, H&E staining and quantitative analysis revealed similar phenomenon in these experimental groups, as evidenced by the restored bone formation of BM-MSCs in the presence of oxidative stress when treated by VC, GF or VC + GF (Figure 8B and 8C).

## DISCUSSION

Many obstacles remain in clinic in the stem cell-based therapies, especially the local oxidative microenvironment, where transplanted MSCs undergo apoptosis. It was noted that up to 80% stem cells died in 2 days after transplantation due to the local adverse microenvironment [33]. At the same time, numerous reports demonstrated that adverse oxidative stress inhibited osteogenesis of MSCs. Therefore, antioxidants are widely applied during cell transplantation in the therapy of bone diseases, such as VC and vitamin E, etc [11, 34–37]. Nevertheless, the efficacy of antioxidants applied is usually limited by their short *in vivo* half-life. Hence, in the present study, we chemically linked VC onto 3D structure of GF, aiming to prolong the half-life of VC. Meanwhile, graphene and its derivatives, including GF, have emerged as a promising nanomaterial in regenerative medicine and tissue engineering since their initial report in 2004 [15], due to their excellent physical and chemical properties [38, 39]. There are numerous studies demonstrating that graphene-based nanomaterials exhibit good biocompatibility and little cytotoxicity in various cell types [38]. In our current study, we employed 3D GFs loaded with VC to promote the growth and differentiation of BM-MSCs, in order to evaluate its efficacy as stem cell therapy based on BM-MSCs. The 3D GFs exhibited excellent biocompatibility. Within 10 hours of seeding, the human BM-MSCs were able to steadily attach to the 3D GFs, with some adhered to the surfaces of culture dishes, leaving nearly no cells floating free in the culture (data not shown). Moreover, we didn't observe any cytotoxicity from 3D GFs in the cultured human BM-MSCs, which was supported by the result that GF rescued H_2_O_2_-impared cell viability. These above observation is consistent with previous results, where 2D graphene films were found to serve as excellent interface material for other types of cells [40–42]. In the preparation of the 3D GF, chemical vapor deposition method was used, which is compatible with high-quality graphene, therefore the biological effect was not severely affected by impurities [19, 40].

Interestingly, GF itself may exhibit the capability against oxidative stress. Qiu *et al.* recently demonstrated the antioxidant property of graphene-based materials, which was attributed to their extreme large surface area to efficiently scavenge free radicals [18]. As the hydroxyl radicals are formed photolytically in the graphene-based materials, their reducing effect is achieved through a synergistic action of inhibition of antioxidant activity inhibition (UV absorption) and scavenging of OH radical. Therefore the 3D GF used in our study also works as an antioxidant nanomaterial, which further protects MSCs from oxidative stress, together with chemically linked antioxidant VC.

In our current study, we have clearly demonstrated that oxidative stress inhibited viability, differentiation and osteogenesis of human BM-MSCs in an *in vitro* cell culture of H_2_O_2_-induced oxidative stress. The application of VC, GF and VC + GF rescued the H_2_O_2_-impaired cell viability and differentiation of human BM-MSCs. Meanwhile, we found that VC, GF and VC + GF restored the H_2_O_2_-inhibited osteogenic process, as evidenced by restored calcium contents and ALP activity, as well as the mRNA levels of both *Osx* and *Runx2* in the cultures. Furthermore, the protective effect of GF, VC and GF + VC against oxidative stress-inhibited osteogenesis was also clearly demonstrated in an *in vivo* mouse model of osteogenesis.

Wnt signaling pathway is reported to be implicated in the osteogenesis of MSCs. In our current study, we have shown that H_2_O_2_ inhibited the Wnt signaling-related regulators, such as cyclin D1 and β-catenin, and such inhibition was rescued by the application of VC, GF and VC + GF, suggesting that Wnt signaling was involved in the H_2_O_2_-impaired osteogenesis, as well as in the following VC or GF-protective effect during osteogenesis. Meanwhile, when the Wnt pathway was inhibited by 0.2 μg/ml DKK-1, the rescue effect of VC, GF and VC + GF was noticeably lower, as evidenced by the reduced ALP activity and calcium contents in the culture, as well as the relative mRNA expressions of *Runx2* and *Osx*. Therefore, it can be reasoned that the Wnt pathway played an essential role in the H_2_O_2_-inhibited osteogenesis and in the antioxidant VC or GF-rescued osteogenesis.

Of clinical significance, we hereby clearly demonstrated that the combination of VC and GF displayed a more potent protective effect against oxidative stress than monotherapy of either VC or GF, in terms of cell viability, differentiation and osteogenesis both *in vitro* and *in vivo*. Our results may shed light on the clinical management of oxidative stress-induced bone-related diseases, and should have certain significance of reference for other oxidative stress-induced diseases. Nevertheless, more careful and systematic examinations on the biosafety of this system should be done before any further clinical use.

## MATERIALS AND METHODS

### GF Synthesis

GFs were synthesized according to previous reports [19–21]. In brief, the chemical vapor deposition method was employed for the synthesis of 3D GFs using Ni foams as template (Alantum Advanced Technology Materials, China). Samples were first immersed into 1.0 M FeCl_3_ solution at room temperature for at least 3 days, then were rinsed sequentially with 1.0, 0.1 and 0.01 M HCl, after which samples were rinsed with running water for at least 3 days for removal of the etching agents.

### Characterization of GFs

The crystallinity of GFs was characterized by Raman spectrometer lamRAM HR800 (HORIBA, France).

### Oxygen plasma treatment

The oxygen plasma treatment was accomplished with a downstream inductively coupled plasma asher (DFS-200, BMR) equipped with a remote oxygen (O_2_) gas. The radio-frequency power to remotely generate plasma was 100 W and the pressure of oxygen was 300 mTorr. After treating the graphene with oxygen plasma, Raman spectroscopy was utilized to characterize the structural changes.

### VC modification

VC modification of graphene was conducted in a sealed bottle with ethanol as medium. The initial concentration of VC in ethanol was 1 M and the pH value was adjusted to 9–10 with 25% ammonia solution. The sealed bottle containing 5 ml VC solution was sonicated in water bath for 10 min and kept at 50°C for 4 h. After that, the modified graphene was rinsed in water for six times to remove the free VC molecules.

### Contact angle

The water drops were dispensed onto the surface of graphene film and then the sessile water contact angle θ was measured on a magnified image captured with a video camera (DataPhysics OCA, Germany).

### Human BM-MSC culture

Human BM-MSCs were isolated and passaged according to previous reports [22, 23]. In brief, 2.0–3.0 ml of bone marrow aspirate were collected from the osteotomy sites of 5 adult healthy donors (24–35 age, two male and three female), who have given written and signed informed consents. The aspirate was then gently mixed with α-minimum essential medium (α-MEM) to make the total volume of 10 ml, which contains 15% embryonic stem cell qualified-fetal bovine serum (ES-FBS) (Gibco, Grand Island, NY, USA), 100 μg/ml streptomycin, 2 mM L-glutamine, 100 U/ml penicillin, 18% Chang B and 2% Chang C (Irvine Scientific, Santa Ana, CA, USA). Isolated cells were then cultured in a humidified incubator at 37°C with 5% CO_2_ for 5 days. Debris and blood were carefully removed by washing with serum-free α-MEM. Cells were then cultured to approximately 90% confluency before being split into fresh medium at 30% confluency. The medium was changed every other day. Cells between passages 3–5 were used for experiments.

For *in vitro* studies, BM-MSCs were initially seeded on the surface of GF at a density of 0.5 × 10^6^, which was placed on the bottom of the 96 well cell culture plates (Corning, NY, USA). After a gentle shake, the drop with BM-MSCs spread into the entire GF scaffold. Then, a final volume of 200 μl culture medium was added. For control and H_2_O_2_ groups, cells were also seeded in the 96 well cell culture plates at the same density.

### 3-(4, 5-dimethylthiazol-2-yl)-2, 5-diphenyl tetrazolium bromide (MTT) assay

MTT assay (Sigma, St. Louis, MO) was employed in the study to assess cell viability. For H_2_O_2_ treatment, cells were incubated with H_2_O_2_ at different concentrations for the first 24 h before changing to fresh culture medium without H_2_O_2_. For VC treatment, VC was present in the culture medium throughout cell culture.

### Osteoblast differentiation

Induction of osteogenic differentiation of human BM-MSCs was performed using 10 nM dexamethasone (Sigma). To examine the osteogenesis, cells were seeded into a 96-well plate at approximately 80% confluency. The culture medium was replaced after 24 h with DMEM osteogenic medium, containing 10% FBS, 60 mM ascorbic acid, 10 mM glyceraldehydes 3 phosphate and 10 nM dexamethasone. The osteogenic medium was changed every other day.

### Flow cytometry

Flow cytometry followed routine protocols. Cells were stained with antibodies specific for CD105, CD29 and CD44, then cells were acquired and analyzed by a BD FACS Canto using the FACS Diva software (BD Bioscience, Bedford, MA, USA). Antibodies used in the study were as follows: CD105-FITC (MCA1557FT, Serotec, Oxford, UK), CD44-FITC (MCA643FA, Serotec), and CD29-FITC (MCA1949FT, Serotec). Fluorochrome-conjugated mouse isotype control IgG (Serotec) was used as negative labeling control.

### Measurement of oxidative stress

ROS, GSH and MDA levels, SOD activity were all determined using commercial detection kits (Jiancheng Biotech, Nanjing, China) following the manufacturer's manuals. Briefly, GSH activity was measured by the reaction of 5.50-dithiobis-2-nitrobenzoic acid (DTNB) with GSH, the product of which can be read at 412 nm using a spectrophotometer. Binding of MDA with thiobarbituric acid results in a chromogenic complex that can be detected at 532 nm. SOD activity can be measured spectrophotometrically based on inhibition reaction of NADPH-phenazinemethosulphate-nitroblue tetrazolium formazan, as enzyme reaction can be detected by recording the absorption at 560 nm. Intracellular ROS level was measured using the 2′,7′-dichlorofluorescin diacetate (DCFH-DA) probe (Sigma) in a detection kit. The ROS level was measured as the absorbance at 525 nm recorded with a spectrometer.

### ALP activity assay

Human BM-MSCs were cultured in osteogenesis medium, which was replaced every other day. After 8 days the cells were washed twice with TB buffer (20 mM Tris, 150 mM NaCl, pH 7.5), and then lysed with 100 μl 0.1% Triton in TB buffer. After centrifugation at 12,000 rpm at 4°C for 20 min, 45 μl supernatant was incubated with 100 μl ALP substrate p-nitrophenyl phosphatate liquid substrate system (Promega, Madison, WI, USA) for 20 min at 37°C, and the absorbance at 405 nm was measured using a spectrophotometer. ALP activity was normalized to total protein.

### Calcium content assay

Human BM-MSCs were cultured in osteogenesis medium with treatments as indicated for 24 days. The medium was changed every other day. On day 24 calcium content in the culture was determined using Calcium Assay kit (Genzyme Diagnostics, Charlottetown, PE, Canada) according to the manufacturer's manual. The absorbance was measured at 650 nm. All samples were measured in triplicates and compared to the standard cure for calcium calibration, and the calcium content was normalized to cell number.

### Quantitative real-time reverse transcription polymerase chain reaction (RT-PCR)

Total cellular RNA was prepared using the RNeasy kit (Qiagen, Valencia, CA), and cDNA was then synthesized using the SuperScript RT III (Invitrogen, Pleasanton, CA), following manufacturer's instructions. RT-PCR was then performed following routine protocols. The primers used for the amplification of target genes were shown in supplementary Table S1. Relative expression levels were normalized to β-actin.

### Immunofluorescence

The cultures were washed twice in PBS buffer, fixed using 4% paraformaldehyde in PBS for 40 min, then blocked and permeabilized in PBS containing 2% BSA and 0.1% triton X-100 for 80 min. Cells were immunostained using primary antibody (β-tubulin, Sigma) for 90 min, washed three times with PBS, followed by incubating with secondary antibody (Invitrogen, USA) for another 70 min. The nucleus was stained with DAPI (Sigma).

### Differentiation of MSCs into myogenic and adipogenic lineages

Human BM-MSCs were cultured for approximately 4 weeks in NM supplemented with 5 μg/ml insulin and 10 nM dexamethasone to induce adipogenic differentiation. The cells were then fixed using 4% paraformaldehyde in PBS at room temperature for 1 h, and stained using Oil Red O (Sigma) solution to confirm their identity. Similarly, cells were incubated with DMEM-H containing 10% FBS, 50 μM hydrocortisone (Pfizer Inc, NY, USA), 5% horse serum (Sigma) and 0.1 μM dexamethasone (Sigma) in order to induce myogenesis. Myogenesis differentiation was observed with crystal violet staining of the formed myotubes.

### Western blot analysis

After treatment, BM-MSCs were washed with PBS for 3 times, followed by incubation with lysis buffer according routine protocols. Primary antibodies used in the study were as follows: cyclin D1 (#2926, Cell Signaling); β-catenin (#13727, Cell Signaling, Boston, MA, USA); GAPDH (#G9545, Sigma). Western blot was quantified using the Image-Proplus 5.0 software (Media Cybernetics Inc., Silver Spring, MD, USA).

### *In vivo* osteogenesis of BM-MSCs

All animal experiments in the study were performed in compliance with the Guide for the Care and Use of Laboratory Animals of NIH. A total of 1 × 10^6^ human BM-MSCs was injected subcutaneously into 8 weeks old male athymic nude mice. After the mice were treated with general anesthesia, small incisions were made in the skin on the back of the mice to form subcutaneous pouches, into which the human BM-MSCs were injected. The skin incisions were then closed using 4–0 nylons. For oxidative stress treatment, the mice were fed with high-fat diet containing 2% cholesterol, 20% coconut oil and 0.125% bile salts, as established previously. [24] VC was orally administrated at 50 μg/Kg body weight. GF or VC + GF was implanted subcutaneously right before BM-MSC transplantation. To measure the calcium contents of *in vivo* implanted specimens, individual samples were deparaffinized, dried at 95°C for 1 h, weighed, and then decalcified in 1 ml Calci-clear Rapid solution. Calcium content of the supernatants was measured using the Methylxylene blue method. H&E staining followed a routine procedure and the histological sections were analyzed by ImageJ software.

### Statistical analysis

All values were are calculated as mean ± S.E.M. All experiments were independently repeated at least 3 times. Student's *t-test* was conducted for comparison between two groups, and *P* < 0.05 was deemed statistically significant unless otherwise noted.

## SUPPLEMENTARY MATERIALS FIGURE AND TABLE


