# Supplementary material for: New strategy to rescue the inhibition of osteogenesis of human bone marrow-derived mesenchymal stem cells under oxidative stress: combination of vitamin C and graphene foams
Source: Oncotarget. 2016 Oct 4;7(44):71998–2010. doi: 10.18632/oncotarget.12456 (PMC5342139; doi:10.18632/oncotarget.12456)
Supplement: Supplementary file 1 [file oncotarget-07-71998-s001.pdf]

New strategy to rescue the inhibition of osteogenesis of human bone marrow-derived mesenchymal stem cells under oxidative stress: combination of vitamin C and graphene foams

Supplementary Materials

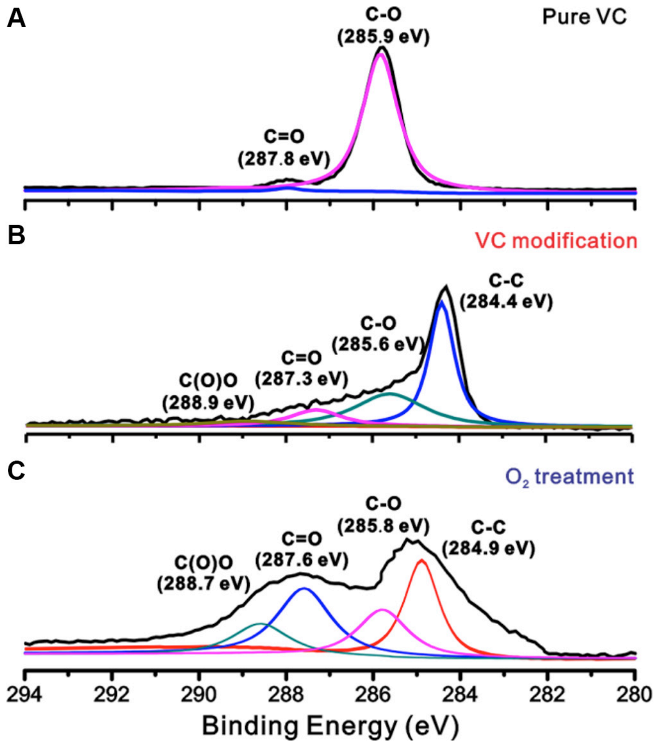

Supplementary Figure S1: High-resolution C (1s) XPS of Pure VC, VC modified GF and O_2 plasma-treated GF. The X-ray photoelectron spectroscopy (XPS) (Axis Ultra DLD, Kratos) was measured by utilizing an Al Kα X-ray source operated at 40 eV.

Supplementary Table S1: Primers for RT-PCR in this study

genes	forward	reverse
Runx2	5′-TCTTAGAACAAATTCTGCCCTTT	5′-TGCTTTGGTCTTGAAATCACA
Osx	5′-CCTCCTCAGCTCACCTTCTC	5′-TTGGGAGCCCAAATAGAAA
Sox-2	5′-GCCTGGGCGCCGAGTGGA	5′-GGGCGAGCCGTTCATGTAGGTCTG
oct-4	5′-AGGTGTTCAGCCAAACGACC	5′-TGATCGTTTGCCCTTCTGGC
nanog	5′-ATCCAGCTTGTCCCCAAAG	5′-ATTTCATTCGCTGGTTCTGG
β-actin	5′-CAATGTGGCCGAGGACTTTG	5′-CATTCTCCTTAGAGAGAAGTGG
